# Mechanical Unloading by Fulminant Myocarditis: LV-IMPELLA, ECMELLA, BI-PELLA, and PROPELLA Concepts

**DOI:** 10.1007/s12265-018-9820-2

**Published:** 2018-08-06

**Authors:** Carsten Tschöpe, Sophie Van Linthout, Oliver Klein, Thomas Mairinger, Florian Krackhardt, Evgenij V. Potapov, Gunther Schmidt, Daniel Burkhoff, Burkert Pieske, Frank Spillmann

**Affiliations:** 1 Charité, University Medicine Berlin, Department of Cardiology, Campus Virchow Klinikum, Berlin, Germany; 2 Charité, University Medicine Berlin, Berlin-Brandenburg Center for Regenerative Therapy (BCRT), Campus Virchow Klinikum, Berlin, Germany; 3 Deutsches Zentrum für Herz Kreislauf Forschung (DZHK) - Standort Berlin/Charité, Berlin, Germany; 4 Helios Klinikum, Berlin, Germany; 5 Department of Cardiothoracic and Vascular Surgery, Deutsches Herzzentrum Berlin (DHZB), Berlin, Germany; 6 Cardiovascular Research Foundation, New York, NY USA; 7 Department of Cardiology, Deutsches Herzzentrum Berlin (DHZB), Berlin, Germany

**Keywords:** Mechanical circulatory support, Mechanical unloading, Fulminant myocarditis, Weaning, Endomyocardial biopsies, MALDI-imaging mass spectrometry, Metabolism

## Abstract

Mechanical circulatory support (MCS) is often required to stabilize patients with acute fulminant myocarditis with cardiogenic shock. This review gives an overview of the successful use of left-sided Impella in the setting of fulminant myocarditis and cardiogenic shock as the sole means of MCS as well as in combination with right ventricular (RV) support devices including extracorporeal life support (ECLS) (ECMELLA) or an Impella RP (BI-PELLA). It further provides evidence from endomyocardial biopsies that in addition to giving adequate support, LV unloading by Impella exhibits disease-modifying effects important for myocardial recovery (i.e., bridge-to-recovery) achieved by this newly termed “prolonged Impella” (PROPELLA) concept in which LV-IMPELLA 5.0, implanted via an axillary approach, provides support in awake, mobilized patients for several weeks. Finally, this review addresses the question of how to define the appropriate time point for weaning strategies and for changing or discontinuing unloading in fulminant myocarditis.

**Electronic supplementary material:**

The online version of this article (10.1007/s12265-018-9820-2) contains supplementary material, which is available to authorized users.

## Introduction

The diagnosis and treatment of myocarditis is still a clinical challenge due to the variability of its clinical presentation ranging from mild dyspnea or chest pain to cardiogenic shock and death [1]. In patients presenting with profound cardiogenic shock, immediate mechanical circulatory support (MCS) is often required to treat the hemodynamic compromise, allowing time for making the proper diagnosis and for initiating anti-inflammatory strategies [2]. Evidence from different registries illustrates that among the various short-term MCS options, fulminant myocarditis patients are most frequently treated with veno-arterial (v.a.) extracorporeal life support (ECLS) [3–6]. However, the use of ECLS increases the afterload of the left ventricle (LV), which, without employing an additional LV venting strategy, can also cause LV distention and exacerbate pulmonary edema. Less well appreciated is the fact that such increases in load with accompanying increases in myocardial wall stress lead to activation of cardiac mechano-transduction pathways, which, over time, induce inflammatory reactions. The combination of increased load and inflammation (which increases extracellular matrix turnover) promotes unfavorable cardiac remodeling. Particularly in an inflammatory disorder, such as myocarditis, a therapeutic strategy is required that, in addition to providing adequate circulatory support, “unloads” the LV, reduces wall stress, and subsequently reduces inflammatory responses [7]. All these goals can be achieved via transcutaneously deployed axial flow pumps like the Impella systems (2.5, CP, 5.0, and RP). The Impella 2.5, CP, and 5.0 devices are miniature axial flow pumps that directly pump blood from the LV to the ascending aorta just above the aortic valve. This approach directly unloads the LV throughout the cardiac cycle, reducing total mechanical work and myocardial oxygen demand, while lowering wall stress and improving subendocardial coronary blood flow. Similarly, the Impella RP pumps blood from the right atrium to the proximal pulmonary artery for right-sided support, providing similar hemodynamic and metabolic effects to the right ventricle (RV).

In this review, we provide an overview of the successful use of an Impella-based strategy for treatment of fulminant myocarditis and cardiogenic shock as a sole means of supporting the circulation, and in combination with ECLS (ECMO plus Impella: ECMELLA), or in combination with RV-Impella RP (BI-PELLA). Furthermore, we provide new evidence that this “unloading” strategy not only provides the required circulatory support but also provides additional disease-modifying effects important for myocardial recovery (bridge-to-recovery) in these patients. Specifically, we emphasize the importance of prolonged use of the LV-Impella for several weeks in this setting, a newly termed PROPELLA (prolonged Impella) concept. Finally, we also address the important questions of how to define the optimal time points and strategies for initiating weaning, escalating, or discontinuing support in fulminant myocarditis.

### LV-Impella Approach in Acute Fulminant Myocarditis

Several case reports have demonstrated successful short-time use of LV-Impella in patients with fulminant myocarditis in which RV function was not significantly impaired and patients were not in need of biventricular support [8–10]. In these prior cases, LV-Impella was used as sole therapy without combined immunosuppressive therapy. In two cases, endomyocardial biopsies (EMB) were not performed [8, 9], while in another case, positive EMB did reveal immune cell infiltrates [10]. In these cases, Impella was used for 5–7 days until the hemodynamics were stabilized and patients could be weaned [6] or, in one case, were bridged to durable LV mechanical support [9].

### The ECMELLA and BI-PELLA Concepts in Acute Fulminant Myocarditis

**Fig. 1:**
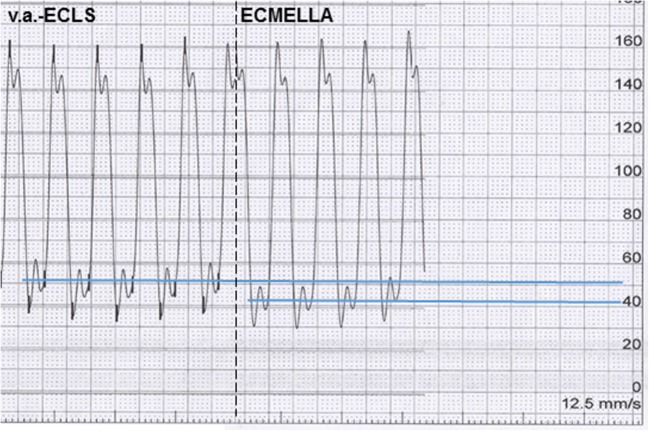
Impella-induced left ventricle venting under ECLS treatment: Concept of ECMELLA—left ventricle unloading. Left panel: invasive LV hemodynamic measurements under veno-arterial (v.a.) extracorporeal life support (ECLS) support indicate highly increased LV end-diastolic pressure (LVEDP) of 51 mmHg. Right panel: invasive LV hemodynamic measurements under ECMELLA concept illustrates immediate lowering of LVEDP to 43 mmHg already 50 s after onset

In cases of fulminant myocarditis with additional severe impairment of the RV, ECLS is the most frequently used form of MCS [3–6]. Nevertheless, mortality of cardiogenic shock despite ECLS is still high in this setting [11]. In fact, such application of ECLS is limited due to the increase of afterload with associated increases in LV filling and pulmonary capillary pressures (Fig. 1, left panel); this increases wall stress and can reduce subendocardial myocardial coronary flow. The increased afterload and filling pressures can be offset by combining ECLS with an LV-Impella device (Fig. 1, right panel), which typically allows the reducing ECLS flow rates. Therefore, it is increasingly recognized that an additional LV unloading strategy can be important for many patients treated with ECLS (movies 3 and 4).

LV venting via cannulation of the left atrium, via transvenous balloon atrial septostomy [12, 13], via atrial stenting [14], or via cannulation of the LV via percutaneous pigtail [15] or larger bore surgically placed cannula via thoracotomy have been successfully performed during ECLS treatment. Other strategies for off-loading the LV during ECLS include intra-aortic balloon pumping (IABP) and the use of inotropes and vasodilators (adequate blood pressure permitting).

A study comprising 135 patients who underwent ECLS and concomitant IABP implantation has shown that ECLS combined with IABP can be an effective therapy in some cases. In that study, prior IABP use was an independent predictor of reduced in-hospital mortality, stroke, or vascular injury [16]. Unfortunately, this study did not include ECLS patients without IABP. Therefore, this study only provides indirect evidence supporting the potential benefit of IABP in addition to ECLS.

Comparing Impella with the abovementioned methods of LV unloading, LV-Impella 2.5 or CP has the advantage to be inserted percutaneously avoiding the need of surgical interventions and provides a higher degree of hemodynamic support compared to IABP. Besides being less invasive than classical decompressive techniques, Impella is associated with lower requirements for blood products with fewer thromboembolic complications [17, 18]. The benefit of this ECMELLA approach to unloading was recently demonstrated in a multi-center retrospective cohort of 157 patients with profound refractory cardiogenic shock compared with patients treated with ECLS alone [19]. Comparison of 42 patients undergoing ECLS alone (control group) with 21 patients treated with the ECMELLA concept revealed that the ECMELLA patients had a significantly lower hospital mortality (47% vs. 80%, *P* < 0.001) and a higher rate of successful bridging to either recovery or further therapy (68% vs. 28%, *P* < 0.001) compared to ECLS patients. This study comprised ischemic as well as non-ischemic-induced cardiogenic shock patients, including myocarditis patients. These promising results need further validation, ideally in randomized studies, in patients with refractory cardiogenic shock. Besides this trial, only a few case reports are available reporting the short-time use of the ECMELLA concept as bridge-to-recovery in fulminant myocarditis [18, 20, 21], indicating the need for further studies in this population.

**Fig. 2:**
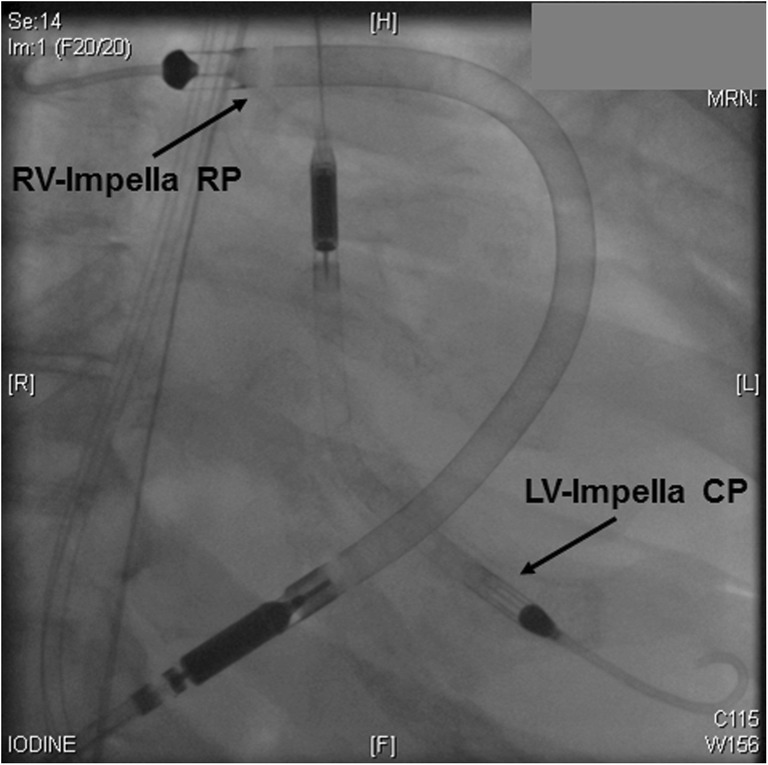
Chest X-ray of a BI-PELLA approach of a patient from our clinic with endomyocardial biopsy-proven severe fulminant myocarditis and biventricular decompensation. RV-Impella RP implanted into the right A. pulmonalis; LV-Impella CP implanted into the left ventricle

As an alternative to the ECMELLA concept to achieve biventricular support, Pappalardo et al. [22] recently reported the first case of a biventricular support with two Impella pumps combining a LV-Impella CP with a RV-Impella RP system for acute biventricular failure due to suspected acute myocarditis. This so-called BI-PELLA concept mitigates the shortcomings of an ECLS increased afterload, while providing percutaneous biventricular unloading and hemodynamic support. This approach fulfills the requirements of the acute MCS concept, which includes (1) ease of access; (2) non-surgical percutaneous insertion [23]; (3) rapid deployment; (4) potent biventricular support; and (5) stepwise weaning of uni- or biventricular support [22], and can be used as a bridge to a durable left ventricular assist device (LVAD) if needed [24]. This concept extends the possibilities of different unloading strategies for patients with biventricular failure in which oxygenation is not a major issue (Fig. 2).

### The Prolonged LV-IMPELLA Concept in the Subacute Phase of Fulminant Myocarditis

As discussed, a strategy that simultaneously provides both sufficient circulatory support and LV unloading can be particularly effective in patients with fulminant myocarditis and cardiogenic shock. However, hemodynamic stabilization alone does not guarantee recovery of myocardial function. Myocarditis is usually characterized by a systemic inflammatory immune system [25–27] which results in a host of myocardial abnormalities including immune cell infiltration [28, 29], cardiac fibrosis [30], dysregulation of titin function [30, 31], and impaired energy metabolism [32].

The impact of prolonged unloading on these myocardial processes and, ultimately, on myocardial recovery in fulminant myocarditis is so far unknown. In the context of chronic heart failure, it is well established that prolonged LV unloading achieved with durable LVADs can lead to reverse remodeling based on anti-fibrotic and anti-inflammatory mechanisms [33, 34], can improve myofilament and titin architecture [35], can reverse deleterious metabolic adaptations of the failing heart, and can even activate cellular pathways of cardio-protection and cardiac repair [36, 37]. These well-established effects of prolonged LV unloading via durable LVAD were the rationale for investigating the impact of prolonged support with an LV-Impella to provide unloading in fulminant myocarditis (the PROPELLA concept).

We report here, for the first time to our knowledge, the impact of a LV-Impella 5.0 implanted through an axillary approach for 39 days combined with standard heart failure therapy (metoprolol, torasemide, valsartan/sacubitril, spironolactone, and ivabradine starting 48 h after Impella implantation; and carvedilol, ivabradine, valsartan/sacubitril, and eplerenone after Impella explantation), which does not represent a causal therapy, and immunosuppressive therapy consisting of prednisolone (starting at 1 mg/kg/day for 4 weeks (at T1: 100 mg; at T3: 90 mg) followed by 10 mg/day weaning all 2 weeks until reaching 10 mg/day maintenance dose), and azathioprine (100 mg/day) in a patient presenting with fulminant myocarditis and cardiogenic shock. With this approach, the patient did not require sedation and was able to be mobilized daily. No catecholamine treatment was needed despite an initial LV ejection fraction of < 10%. Anticoagulation was maintained with intravenous heparin (partial thromboplastin time between 60 and 80 s). Improvements of cardiac function were observed within 5 days of MCS and inotropic support was not needed. Following temporary reduction of pump flow on day 21, LV performance was improved, indicating the ability of the native heart to provide circulatory support (movie 1: full LV-Impella 5.0 support at level P8 and movie 2: LV-Impella 5.0 at level P1). This patient was supported for a total of 39 days and was able to be weaned with a final LV-EF of 62%. Impella weaning was performed without invasive hemodynamic measurement via pulmonary arterial catheter or a PICCO system, but under invasive RR measurement with permanent ECG and peripheral pulse oximetry monitoring, since the patient was already daily mobilized.

**Fig. 3:**
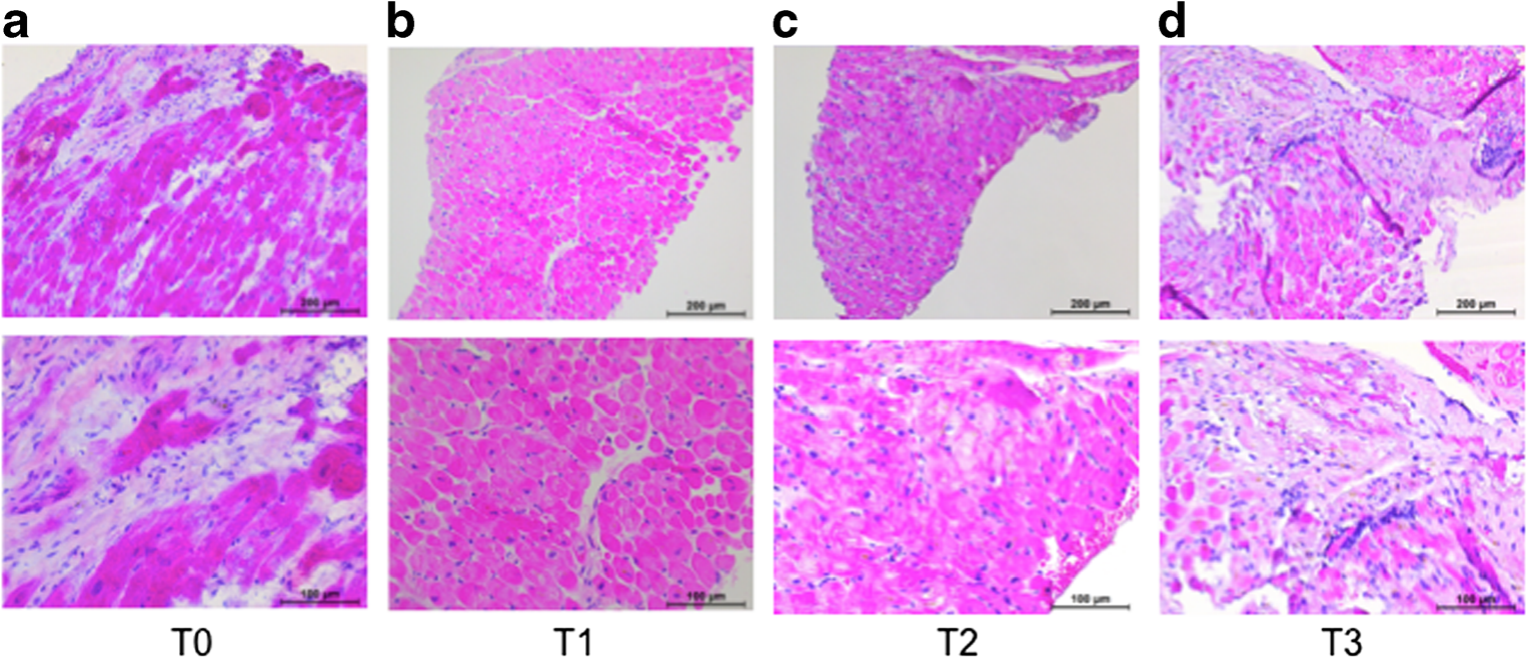
Impact of prolonged mechanical unloading on cardiac immune cell infiltration in chronic inflammatory cardiomyopathy (PROPELLA concept). Upper (× 100 magnification) and lower (× 200 magnification) panels depict hematoxylin and eosin-stained sections of endomyocardial biopsies isolated before (T0), during combined unloading and immune suppression (T1, T2) and after Impella explantation (T3). Unloading combined with immune suppression (T1, T2) decreases the extensive immune cell infiltration found at T0. However, after explantation of the Impella, immune cell infiltration is again prominent (T3)

**Fig. 4:**
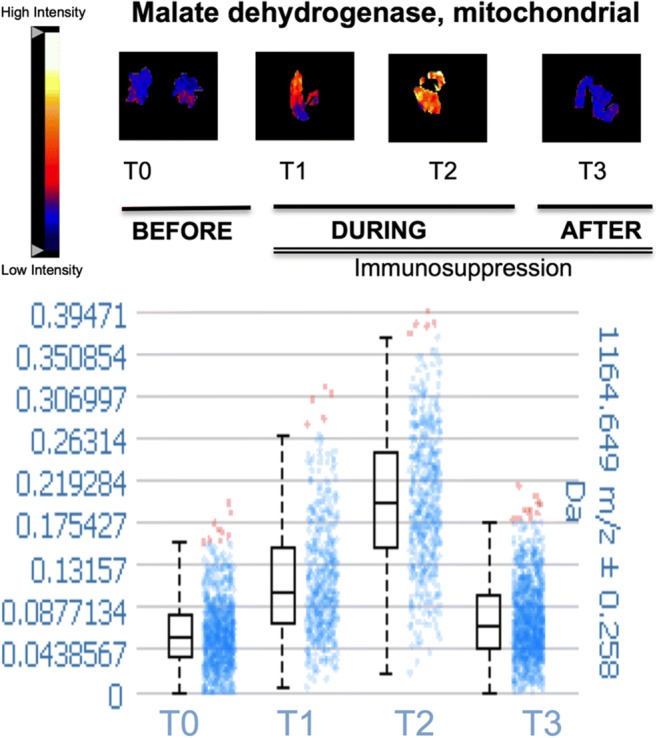
Impact of prolonged mechanical unloading on cardiac metabolism in chronic inflammatory cardiomyopathy (PROPELLA concept). The intensity distribution of malate dehydrogenase, mitochondrial (1164 Da) is significantly increased during combined unloading and immune suppression (T1, T2) compared to before (T0) and after unloading (T3) implantation. Lower panel illustrates the corresponding box plot

In order to gain insights into the impact of MCS and immunotherapy, EMBs [38] were obtained prior to (T0), at two time points during the course of MCS and immunosuppressive therapy (T1 at 17/18 days; T2 at 31/32 days post Impella implantation/immunosuppressive therapy (PROPELLA concept)) and at one time point following withdrawal of support (T3, 3 days following explant). Histological evaluation via hematoxylin and eosin staining revealed that combined MCS and immunosuppressive therapy (T1 and T2) reduced the infiltration of immune cells as observed at T0 (Fig. 3). However, this effect was abrogated after removal of the LV-Impella 5.0 support (T3) despite continuation of immunotherapy, suggesting a primary “unloading”-dependent mechanism. Hypothesis-free analysis via matrix-assisted laser desorption/ionization (MALDI) imaging mass spectrometry [39] further revealed that the LV expression of malate dehydrogenase enzyme (which is a key enzyme involved in the reduction of NAD+ to NADH cycle in the tricarboxylic acid cycle and key step in generation of ATP) was only increased during unloading and immunosuppression and dropped after LV-Impella 5.0 explantation (Fig. 4). Knowing that LV pressure and volume overload alters metabolic substrate utilization, decreases mitochondrial function, and reduces energy production in the failing heart [40] and that the expression of malate dehydrogenase enzyme is reduced in heart failure [41, 42], these observations support the notion that unloading restores the downregulated expression of malate dehydrogenase enzyme in the LV. However, this beneficial effect was abolished after explantation of the LV-Impella 5.0 support. Whether this normalization in malate dehydrogenase enzyme expression represents an improved glucose oxidation or rather an increased anaplerosis flux due to regression of hypertrophy after mechanical unloading, as seen by Diakos et al. [43] in chronic heart failure patients following LVAD, requires further investigation.

### Weaning Under Temporary Mechanical Circulatory Support with Impella

Most cardiac arrest centers perform weaning from ECLS and Impella support without clear guidelines or algorithms [44]. According to our experience, we found dynamic echocardiography-based investigations very helpful to define a suitable time point for weaning and for changing or discontinuing unloading strategies in fulminant myocarditis. In patients with fulminant myocarditis, we found a very unique behavior during unloading support: as shown in movie 1 (PROPELLA concept) and movie 3 (ECMELLA concept), we found a continually reduced LV ejection fraction during full unloading support. But temporally reducing unloading support led to an immediate increase of ejection fraction (movie 2; PROPELLA concept; movie 4; ECMELLA concept) indicating that full unloading induced a form of resting of the LV, which maybe an important component of the mechanisms leading to recovery. This is in contrast to patients with severe myocardial infarction, where temporary pump flow reductions under echocardiographic evaluation could not show any further improvement of LV function (movies 5–8). Thus, temporary pump flow reductions under echocardiographic evaluation are useful in clinical practice for assessing suitability for weaning.

In principle, investigation of the LV and the RV function before PROPELLA or ECMELLA weaning is fundamental to anticipate univentricular or biventricular recovery and the need for possible subsequent LVAD placement. Before weaning, patients under Impella support must meet the criteria of being afebrile and euvolemic and compensated with resolution of pulmonary edema and adequate arterial PaO_2_. Patients must be free of the need for inotropic or pressure support, should have normal physiological parameters (arterial blood pressure, central venous pressure, heart rate, and rhythm), and all other parameters of end organ dysfunction should be recovered to baseline.

In our department, we established a weaning protocol consisting of four stages:

- Stage 1: baseline RV and LV function of the heart are measured by echocardiography on full LV-Impella support.
- Stage 2: after evaluation of baseline data, Impella RPMs (and therefore flow) is decreased in single steps (e.g., P8 to P7, and so on) with the goal of achieving half of the original RPMs with maintenance of adequate hemodynamics. At every flow level, RV and LV function and hemodynamic responses (blood pressure and heart rate) are monitored over 5–10 min to allow estimation of ventricular function and volume status. If, at any period in the weaning protocol, RV or LV distension occurred or significant hypotension or increase in heart rate is observed, the weaning protocol is stopped and Impella support is returned to full flow.
- Stage 3: If stage 2 was successful, Impella support will continue to be reduced by one step for 24 h and then reevaluated like described under stage 2.
- Stage 4: If stable RV and LV function, hemodynamics and volume status are maintained for 48 h on P2 Impella support, inotrope stress test with dobutamine will be performed in which RV and LV functions and hemodynamics are observed for responses over 30 min. If both, RV and LV function are recovered, the patient will be considered for Impella removal. This procedure is not performed in patients who spontaneously increase their LV function during pump reduction flow, as is often detectable in myocarditis patients with recovery. Here, we decide to support unloading as long as possible to be able to invoke the proposed disease-modifying mechanism for complete recovery (PROPELLA concept). In our experience, a time frame of about 4 weeks is appropriate for this purpose, since the weaning stress test was positively completed. We also wanted to reduce potential side effects like embolism and infections under immunosuppression. Therefore, we found the explantation time point after 4 weeks in this case appropriate. However, the explantation time point can differ depending on the individual clinical scenario.

## Conclusion

There is accumulating evidence showing that LV unloading via a transcutaneously placed axial flow pump is a viable treatment option for patients with fulminant myocarditis and cardiogenic shock. Such therapy is feasible as sole LV MCS when RV function is sufficient, but can be used in combination with ECLS (the ECMELLA concept) or in combination with a right-sided Impella RP (the Bi-PELLA concept). One of the advantages of the ECMELLA approach is the possibility to deescalate the ECLS flow rates and reduce loading effects on the LV. Improvement of RV function can permit removal of the more invasive ECLS approach, enabling the conversion of the ECMELLA to the PROPELLA concept, if longer LV hemodynamic support is still necessary.

Besides circulatory support and LV decompression, of which the latter is required to reduce myocardial wall stress, decrease myocardial oxygen requirements, and enhance the chances of recovery, we report for the first time that prolonged unloading (the PROPELLA concept) leads to additional disease-modifying effects over time that can be important for enhancing myocardial recovery in patients with chronic fulminant myocarditis. These disease-altering effects include unloading-induced reductions of myocardial inflammation, modulation of cardiac remodeling, and restoration of more normal metabolic machinery. Such effects may be critical to the restoration of cardiac structure and function, suggesting that the therapeutic effects of Impella support go beyond its primary use as mechanic support for normalizing hemodynamics. Evidence illustrating the impact of LV unloading on cellular and molecular mechanisms influencing cardiac remodeling, fibrosis, inflammation, and calcium metabolism is so far mainly derived from studies of durable LVADs [45, 46]. Mechanistic data demonstrating the effect of LV unloading on fulminant myocarditis are lacking. Particularly, in an inflammatory disorder, such as myocarditis, additional research is needed into how LV unloading and decreased wall stress might affect inflammatory responses [7], and consequently may contribute to mitigating its long-term consequences. The heterogeneity of the presentation and clinical course of fulminant myocarditis makes it difficult to determine the appropriate time for discontinuing Impella support or converting to a ventricular assist device. EMB analysis providing information about the status of inflammation, molecular abnormalities, and changes in metabolic processes [47] may therefore provide markers helpful in determining when myocardial recovery is sufficient to warrant weaning. Larger scale clinical trials—if possible including EMB analysis—will help validate these promising concepts, which will bring new light on the use and duration of unloading as a treatment option for chronic fulminant myocarditis.

## Electronic supplementary material

Movie 1 Temporary pump flow reduction maneuver under echocardiographic evaluation in a patient with fulminant myocarditis and a prolonged Impella 5.0 support (PROPELLA concept) at day 21. **A** (movie 1): Full Impella 5.0 support at level P8: 5.2 L/min and severe reduced LV function. (MP4 871 kb)

Movie 2 Temporary pump flow reduction maneuver under echocardiographic evaluation in a patient with fulminant myocarditis and a prolonged Impella 5.0 support (PROPELLA concept) at day 21. **B** (movie 2): Temporary reduction of Impella 5.0 support at level P1: 1.2 L/min, showing an immediate increase in LV function indicating that reduced LV function during full circulatory support (movie 1) belongs to a beneficial LV unloading mechanism and can be used as a sign for recovery. A temporary pump flow reduction maneuver under echocardiographic evaluation is useful in clinical practice for assessing suitability for weaning of the PROPELLA-concept. (MP4 786 kb)

Movie 3 Temporary pump flow reduction maneuver under echocardiographic evaluation in a patient with cardiogenic shock based on a fulminant myocarditis and maximal mechanical circulatory support with ECLS and Impella (ECMELLA concept) at day 7. **A** (movie 3): Reduced ECMELLA support (ECLS flow 2.4 L/min, Impella CP at level P6: 2.4 L/min showing reduced LV function. (MP4 873 kb)

Movie 4 Temporary pump flow reduction maneuver under echocardiographic evaluation in a patient with cardiogenic shock based on a fulminant myocarditis and maximal mechanical circulatory support with ECLS and Impella (ECMELLA concept) at day 7. **B** (movie 4): Temporary reduction of ECLS flow 1.8 L/min and Impella CP at level P1: 0.8 L/min, showing an immediate increase in LV function indicating that reduced LV function during higher circulatory support (movie 3) belongs to a beneficial unloading mechanism and can be used as a sign for recovery. A temporary pump flow reduction maneuver under echocardiographic evaluation is useful in clinical practice for assessing suitability for weaning of the ECMELLA-concept. (MP4 889 kb)

Movie 5 Temporary pump flow reduction maneuver under echocardiographic evaluation in a patient with ischemic cardiomyopathy at day 7. **A** (movie 5): Full Impella CP support at level P8: 3.4 L/min and severe reduced LV function. (MP4 1195 kb)

Movie 6 Temporary pump flow reduction maneuver under echocardiographic evaluation in a patient with ischemic cardiomyopathy at day 7. **B** (movie 6): Temporary reduction of Impella CP support at level P1: 1.2 L/min, showingno change in LV function, indicating that no LV function recovery can be expected at this time point. (MP4 1119 kb)

Movie 7 Temporary pump flow reduction maneuver under echocardiographic evaluation in a patient with cardiogenic shock based on an acute myocardial infarction under maximal mechanical circulatory support with ECLS and Impella (ECMELLA concept) at day 8. **A** (movie 7): Reduced ECMELLA support (ECLS flow 2.0 L/min, Impella CP at level P6: 2.4 L/min showing severe reduced LV function. (MP4 776 kb)

Movie 8 Temporary pump flow reduction maneuver under echocardiographic evaluation in a patient with cardiogenic shock based on an acute myocardial infarction under maximal mechanical circulatory support with ECLS and Impella (ECMELLA concept) at day 8. **B** (movie 8): Temporary reduction of Impella CP at level P1: 0.8 L/min under reduced ECLS flow of 1.0 L/min, showing a close to comparable LV function, indicating that no LV function recovery can be expected at this time point. (MP4 347 kb)

## Abbreviations

BI-PELLA: Combination of LV-Impella and RV-Impella RP
ECLS: Extracorporeal life support
ECMELLA: Combination of ECMO and LV-Impella
EMB: Endomyocardial biopsy
IABP: Intra-aortic balloon pump
LV: Left ventricle
LVAD: Left ventricular assist device
LVEDP: Left ventricular end-diastolic pressure
MCS: Mechanical circulatory support
PROPELLA: Prolonged LV-Impella
RV: Right ventricle
v.a.: Veno-arterial
